# Research on the genetic control of flowering in potato set to blossom

**DOI:** 10.1093/jxb/erz544

**Published:** 2020-01-23

**Authors:** Nigel G Halford

**Affiliations:** Department of Plant Sciences, Rothamsted Research, Harpenden, Hertfordshire, UK

**Keywords:** Flowering control, hybrid potatoes, potato, *Solanum tuberosum*

## Abstract

This article comments on:

**Seibert T, Abel C, Wahl V**. 2020. Flowering time and the identification of floral marker genes in *Solanum tuberosum* ssp. *andigena*. Journal of Experimental Botany 71, 986–996.

**‘Hello, just inspecting my spuds and spotted this fruit growing on a stem. It’s dark green and has a stalk attached. Anyone know what it might be?’ This was a question submitted by a gardener to an online discussion forum (Gardener’s World, 2014), and it is not unusual for gardeners to ask why their potato plants have flowers or berries (often described as small green tomatoes) on them (Fig. 1). The common misconception that potatoes do not flower or produce seeds arises, of course, because potatoes reproduce vegetatively as well as sexually, and growers plant ‘seed’ potatoes (pieces of tuber) rather than true seeds.**

**Fig. 1.**
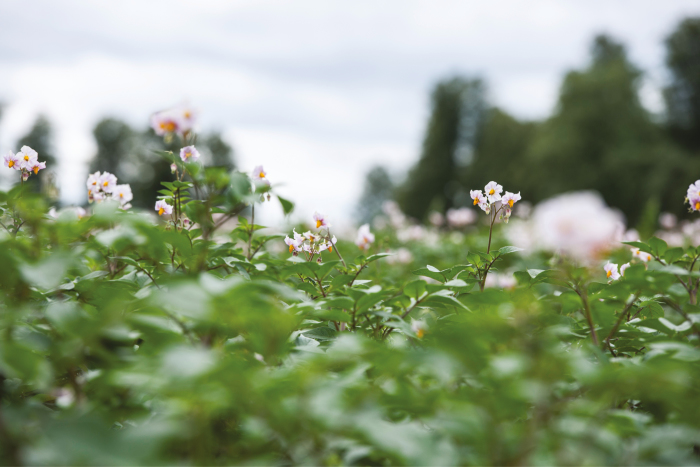
Potatoes flowering in plots at Rothamsted Research in the UK (Graham Shephard, Rothamsted Research).

The potato (*Solanum tuberosum*) originates from Central and South America, of course, and the first plants brought to Europe in the 16th century were of the *andigena* subspecies (*Solanum tuberosum* ssp. *andigena*) from the Andean Altiplano (high plain) of west-central South America. As such, they were adapted to conditions in which day length did not change much through the year and, when grown in the higher latitudes of Europe, would not tuberize until the autumn, when day length had reduced to ~12 h. This made the potato difficult to cultivate in higher latitudes, although by the early 19th century it was already a staple in countries such as Ireland where the mild climate was conducive. Efforts by breeders to develop varieties that would tuberize earlier in the season under European and North American conditions were accelerated by the incorporation of genotypes from Chile in the mid-19th century, including the wonderfully named Rough Purple Chili potato (Bethke *et al.*, 2014). These genotypes were of the subspecies *tuberosum* (*Solanum tuberosum* ssp. *tuberosum*) and enabled the development of early-maturing varieties such as Garnet Chili, Early Rose, and Burbank, the last being selected by the most famous plant breeder of the time, Luther Burbank (1849–1926). These varieties were produced by sexual crossing, but potato is an outbreeder, with most genotypes strictly self-incompatible, so once a suitable genotype had been selected from a cross it would be bulked up vegetatively, and genetically identical ‘seed’ potatoes would be sold to farmers for cultivation. Luther Burbank subsequently selected and multiplied a mutant of Burbank that was originally marketed in 1902 as Netted Gem (Bethke *et al.*, 2014). An alternative name of Russet Burbank was gradually adopted and the variety came to dominate US potato production. Favoured by the fast-food industry for making French fries because of its high dry matter content and convenient, oblong shape, the Russet Burbank is still grown today. Similarly, the method of keeping a variety genetically pure by bulking it up vegetatively and supplying farmers with ‘seed’ potatoes rather than true seed is still used by breeders in most parts of the world, with true seed being used only by subsistence farmers in parts of Africa, Asia, and South America.

If genetic uniformity is the great advantage of vegetative propagation, there are disadvantages associated with it as well. Seed potatoes are much more likely than true seeds to carry diseases, and are bulky, heavy, and expensive to transport. Perhaps the biggest disadvantage is that breeding new varieties is painfully slow, with the process of crossing, selection, and vegetative multiplication taking many years, with relatively modest genetic gains at the end. As a result, there has been little improvement in potato yield in a century (Douches *et al.*, 1996); indeed, there has been little improvement at all in French fry varieties since Russet Burbank was launched, which explains why that variety is still competitive today.

The intractability of potato breeding has also made it difficult to introgress disease resistance genes from wild relatives into breeding programmes; something made all the harder by the fact that *S. tuberosum* is a tetraploid while many of its wild relatives are diploids. This is particularly important because European and North American varieties were initially bred in the absence of late blight, the devastating disease caused by the oomycete, *Phytophthora infestans*, and resistance was lost. In recent years, breeders have used genetic modification to move resistance genes from wild potato species into their breeding lines, with some success (USDA-APHIS, 2014), but that option remains unavailable to European breeders.

The use of vegetative propagation and ‘seed’ potatoes has also meant that potato growers have not been able to benefit from the availability of F_1_ hybrids. Hybrid breeding began in earnest in the 1930s with maize (*Zea mays*): inbred lines would be crossed to exploit the phenomenon of heterosis, in which hybrid offspring would outperform either parent due to the combination of alleles they inherited, with the effects of ‘poor’ alleles in each parent being reduced in the hybrid offspring. The technique has been widely used in plant breeding for many crop species for decades, but in potato it has been made impossible by self-incompatibility and inbreeding depression. That may be about to change, because, in 2011, it was reported that the company Solynta, a Wageningen University spin-off, had overcome these limitations. This was achieved by introducing a self-compatibility restorer gene, *Sli*, from the wild species *Solanum chacoense* into diploid germplasm from the Wageningen University potato breeding programme and developing inbred, self-compatible genotypes (Lindhout *et al.*, 2011). This potentially disruptive innovation showed that breeding hybrid potatoes and distributing seed rather than ‘seed’ potatoes could be feasible (Lindhout *et al.*, 2018).

The prospect of hybrid potatoes and breeders marketing true potato seeds rather than ‘seed’ potatoes means that the genetic control of potato flowering is suddenly very much in focus. This is what makes the study by Tanja Seibert, Christin Abel, and Vanessa Wahl at the Max Plank Institute of Molecular Plant Physiology in Potsdam, published in this issue of the *Journal of Experimental Botany*, so important (Seibert *et al.*, 2020). The team worked on ssp. *andigena*, in which, as described above, tuberization is only induced under short-day conditions, and studied flowering time under long days, so that it would not be affected by tuberization. They developed an optimized growth protocol involving tissue culture followed by cultivation in soil, ensuring that they had uniformly growing plants in which the time frame for floral induction was extremely consistent. This enabled them to obtain exquisite images of the morphological changes that occurred as the shoot apical meristem transitioned from a vegetative to a reproductive state. An important observation was that this transition began with the meristem increasing in size and becoming dome shaped, 10–14 d before the appearance of flower buds and anthesis.

The team also identified marker genes for the floral transition and flower organ development, drawing on knowledge generated in the model plant, Arabidopsis (*Arabidopsis thaliana*), and the model fruit plant, tomato (*Solanum lycopersicum*) (Jaeger *et al.*, 2006; Amasino and Michaels, 2010). Tomato, like potato, is a member of the *Solanum* genus, but since it is the fruits (or berries) that are eaten, flowering control has a direct effect on yield and has been studied extensively (see, for example, Lifschitz and Eshed, 2006). Not surprisingly, perhaps, more similarities were seen with the tomato than with the Arabidopsis system, and the team showed that *WUSCHEL HOMEOBOX 9* (*WOX9*), which encodes a transcription factor, was specifically expressed in the inflorescence meristem, as it is in tomato (Lippman *et al.*, 2008), while *ANANTHA*, which encodes an F-box protein that is exclusively expressed in tomato flower organs, was a marker for the flower meristem. While there were some differences in the gene expression of putative flowering regulators compared with their Arabidopsis homologues, the expression patterns of the MADS box transcription factor gene *SUPPRESSOR OF OVEREXPRESSION OF CONSTANS1* (*SOC1*) and the bZIP transcription factor gene *FD* suggested that these genes might have similar roles to their Arabidopsis counterparts in regulating flower development.

The protocols developed in the study and the results obtained will aid other researchers working on potato flowering and, although they are still in development, if hybrid potatoes fulfil their promise we are likely to see a lot more research on the genetic control of potato flowering in the future.
